# Network Pharmacology-Based identification of pharmacological mechanism of SQFZ injection in combination with Docetaxel on lung cancer

**DOI:** 10.1038/s41598-019-40954-3

**Published:** 2019-03-14

**Authors:** Tan Li, Zhu Baochen, Zhang Yue, Wang Cheng, Wu Yali, Sun Zongxi, Zhang Wantong, Lu Yang, Du Shouying

**Affiliations:** 1 Beijing University of Chinese Medicine, Beijing, 100029 China; 2 Livzon Pharmaceutical Group Inc., Zhuhai, 519020 China; 3 China Academy of Chinese Medicine Sciences, Xiyuan hospital, Beijing, 100091 China

## Abstract

Docetaxel is the widely-used first-line therapy to treat lung cancer around the world. However, tumor progression and severe side effect occurred in some patients with docetaxel treatment. Most of the side effects were caused by immunocompromise, which limits the long-term use of docetaxel. Shenqi Fuzheng (SQFZ) injection has been used as adjuvant therapy to treat lung cancer which may enhance immunity as well. Owing to the complexity of drug combination, the mechanism of SQFZ injection in combination with docetaxel on lung cancer remains unclear. Therefore, a network pharmacology-based strategy was proposed in this study to help solve this problem. Network pharmacology approach comprising multiple components, candidate targets of component and therapeutic targets, has been used in this study. Also, *in vivo* and *in vitro* experiment was applied to verify the predicted targets from network pharmacology We established mouse lung cancer model and inject with docetaxel and SQFZ injection. Tumour weight, spleen index, thymus index, immunohistochemical staining and ELISA were conducted to evaluate the effect and underlying mechanisms of docetaxel and SQFZ injection. Besides A549 cells were also administrated by docetaxel and SQFZ.The indexes BCL2, CASP3 and CASP9 were determined after administration. The results indicated that combination of SQFZ and docetaxel could reduce tumour weight, enhance the spleen index, thymus index. Meanwhile, it could improve the activity of caspase-3 and IL-2 in mice and caspase-3, caspase-9 in A549 cell and inhibit the activity of BCL-2 in A549 cell, which verified the potential protective targets predicted by network pharmacology. In conclusion, combination of SQFZ and docetaxel could increase the curative effect by inducing tumour to apoptosis and play a key role on immunoprotection to reduce side effects.

## Introduction

Lung cancer is one of the most common malignancies in the world with survival period less than 5 years^1^. Over 90% patients with lung cancer need chemotherapy. Docetaxel,an analogue of paclitaxelis^2,3^ is the standard first-line chemotherapy drug which presents an obvious effect in clinic^4,5^. The main pharmacological mechanism of docetaxel is binding tubular protein, and making cells stay in the G2/M phase^6^. It also has the effect of anti-tumor angiogenesis and apoptosis^7,8^. However, tumor progression still happen in some patients through the treatment process of docetaxel^9^. Serious side effects even might occur after using docetaxel, such as anaphylaxis, gastrointestinal reaction, weakness, edema and so on. Thus, finding new therapeutic strategy for lung cancer turns to be important.

Traditional Chinese medicine(TCM) plays an increasingly important role in treating cancer. Many clinical cases showed that Chinese herbal medicine, used as auxiliary drug, have good anti-tumour effect. Shenqi Fuzheng (SQFZ) injection is widely used in clinic as an adjuvant therapy in treating lung cancer, esophagus cancer and colon cancer^10–12^. According to some results of clinical trials, it can enhance the efficacy combining with chemotherapy drugs, with an improvement of immune function and life quality^13^. It has reported that in combination with SQFZ injection, carboplatin chemotherapy’s gastrointestinal reaction and blood toxicity are reduced^14^, which shows it could be a promising drug in combination with docetaxel. Previous research of our lab shows that SQFZ injection alone has no anti-cancer effect. However, when in combination with docetaxel, SQFZ injection could enhance docetaxel’s efficacy on A549 and Lewis lung cancer cell^15^. But the mechanism is still unclear.

Network pharmacology is a new discipline based on the characteristics of biological molecules. It based on multiple authoritative databases which allows us to form an initial understanding of the mechanisms of medicine and diseases. Network pharmacology have been used in our previous research which provide a promising approach to study the pharmacological mechanism of Chinese medicine^16^. In this study, we develop a network pharmacology analysis to identify SQFZ injection’s mechanism in anti-cancer as well as drug-drug interaction with docetaxel. Then conduct *in vivo* and *in vitro* experiment based on the results of network pharmacology analysis.

## Methods

### Determination of Ingredients of SQFZ injection

The main Ingredient of SQFZ injection has reported in several research with various conclusion^17–21^. In this part, the ingredients in these results were gathered and searched in the database to achieve the molecular mass. And SQFZ injection was identified with UPLC-MS. By comparing molecular mass in database with our experiment we verified the ingredients in SQFZ injection and use these ingredients to conduct the network analysis.

1. UPLC-MS: The condition of UPLC-MS (LTQ-Orbitrap XL,Thermo scientific, USA) was as follow. The ESI source was set in both positive and negative ionization mode, respectively. With the capillary voltage set at 3500 V. The mobile phase is as follow: acetonitrile: 0.1% phosphoric acid (0–15 min 5:95–30:60, 15–30 min 30:60–95:5) pumping at a flow rate of 1.0 mL/min. The injected volume was 3 μL.
2. The results of other researches were gathered and were compared with relative molecular mass of ingredients in astragalus and codonopsis pilosula from TCMSP database (http://lsp.nwu.edu.cn/tcmsp.php). Then the molecular mass were compared with UPLC-MS results using Xcalibur 2.2.0 software. The main ingredients in SQFZ injection were collected.

### Candidate targets of drugs

The candidate targets of docetaxel and SQFZ injection were obtained from Genecards Databases (http://www.genecards.org/). After filtering out low correlative targets (relevance score <1), a total of 452 targets of Docetaxel, 6 of 5-Hydroxymethylfurfural, 2 of syringin, 23 of astragaloside, 52 of formononetin, 9 of calycosin, 76 of vernine, 2 of coniferin, 29 of vanillic acid were obtained. After removing duplicate values, a total of 182 candidate targets of SQFZ injection were collected.

### Known therapeutic targets in the treatment of lung cancer

The known therapeutic targets in lung cancer were also acquired from TTD database (http://bidd.nus.edu.sg/BIDD-Databases/TTD/TTD.asp) and genecards database (http://www.genecards.org/). After filtering out low correlative targets (relevance score <1) and removing duplicate values, a total of 2533 targets related to lung cancer were collected.

### Network construction and analysis

Network construction was made by Cytoscape software (http://www.cytoscape.org/). The candidate compound-candidate target network (cC-cT network) was build by linking the compounds to their targets. Compound-potential target-disease network (C-pT-D network) was established by connecting the potential targets with the compounds and lung cancerthe nodes of targets were order by their relevance score in databases.

### Pharmacological verification of network analysis

#### *In Vivo* Experiment

Establishment of subcutaneous lung cancer model: Male C57BL/6 mice (18–22 g, supplied by Vital River Laboratory Animal Technology Co. Ltd. (Beijing, People’s Republic of China)) were acclimatized for 3 days before experiment. Mixed the mouse Louis lung cancer cells (LLC cells,the cell concentration is 1 × 10^6^ · ml^−1^) with Matrigel matrix glue and loaded into 80 liters/unit insulin syringe. Anesthetized and fixed the mice. After disinfection, pre-prepared cell suspension was injected into left axillary region of mice.Normally fed for 2 weeks.

Animal treatment and tissue preparation: C57BL/6 mice were housed in the specific-pathogen-free facility at laboratory of Beijing University of Chinese Medicine. All the experiments on animals were performed under the Guidelines for the Care and Use of Laboratory Animals. The protocols were approved by the institutional animal experimentation committee of Beijing University of Chinese Medicine.

The successful model mice were randomly divided into three groups (control group, docetaxel group, and SQFZ + docetaxel group) and each group had 5 mice. Docetaxel group were injected with docetaxel injection(Newbio Pharm-tech, Wuhan, China)at 18:00 every 3 days, SQFZ + docetaxel group were injected with SQFZ injection(Livzon corporation, China) at 8:00 every day and docetaxel injection at 18:00 every 3 days.

Mice were weighed after 19days’ administration. Then mice were executed 24 hours after the last administration, shaped tumor, spleen and thymus were taken and weighed.

Immunohistochemical Staining: Segments of tumour were fixed on a 4% paraformaldehyde solution for 48 hours and embedded in paraffin. Samples were cut, deparaffinized and hydrated. Then Immunohistochemical staining was done for the evaluation of apoptosis status via caspase-3 antibody (Cell Signaling, Danvers, MA, USA). The histology of tumour were evaluated by microscope (BX53; Olympus Corporation, Tokyo, Japan). The average optical density of images was analyzed with Image-Pro Plus software.

Enzyme-Linked Immunosorbent Assays (ELISA): IL-2 activity of mice blood was measured according to manufacturer recommendations of ELISA kit. Blood of mice was taken and centrifugated. 100 μL serum of protein was used. Each sample was added in 2 wells, take the average for statistical analysis. IL-2 activity was measured by a pan-wavelength micro plate reader (Multiskan GO, Finland).

#### *In Vitro* Experiment

Cell Treatment: A549 cells (human pulmonary carcinoma cell, supplied by Academy of Military Medical Sciences, Beijing, China)were cultured in DMEM with 10% heat-inactivated fetal bovine serum (FBS) as well as 100 U/ml penicillin and 100 μg/ml streptomycin (Thermo Fisher Scientific). Then were seeded in 6 well plates with a density of 1 × 10^5^ cells/mL, 2 ml each well. Incubated for 24 h in 37 °C, 5%CO_2_.

A549 cells were randomly divided into three groups (control group, docetaxel group, SQFZ group and docetaxel + SQFZ group). Control group were administrated with regular culture medium; docetaxel group was administrated with medium of 0.8 μg/L docetaxel; SQFZ group were administrated with medium of 0.1 ml/L SQFZ injection; docetaxel + SQFZ group were administrated with medium of 0.1 ml/L SQFZ injection and 0.8 μg/L docetaxel. Incubated for another 24 h in 37 °C, 5%CO_2_. The medium was discard after 24 h. Cells were washed 2 times with PBS. Then 400 μL cell lysis buffer was added into each well. After mixing with cell, the buffer was placed in 95 °C for 10 min,then cool with ice. Supernatant was taken after 5 min’s centrifuge (10000 r/min),stored in −80 °C for western blot analysis.

Western Blot Analysis: Anti-bcl2, anti-caspase-3 and anti-caspase-9 were obtained from Cell Signaling (Danvers, MA, USA). Membranes were incubated at 4 °C overnight with the specified primary antibody: mouse anti-human protein monoclonal antibody 5D2 (Cell Signaling Technology, USA) and GAPDH (mouse, 1:500). After being washed three times with PBS-Tween 20 (Biodee Biotechnology, Beijing, China), the membranes were incubated with goat anti-mouse IgG-HRP (Cell Signaling Technology, USA). Then membranes were scanned using an Odyssey Imaging System (LI-COR Bioscience, Lincoln, NE). Western blot bands were measured using the ImageJ software and normalized using GAPDH as a control. The results of western blot analysis were valued via ImageJ software. Statistics.

Statistical analysis was carried out by SAS9.3. The protein activity of each group was compared using t-Test and ANOVA with statistical significance set at p < 0.05.

## Results

### Determination of ingredient of SQFZ injection

**Figure 1:**
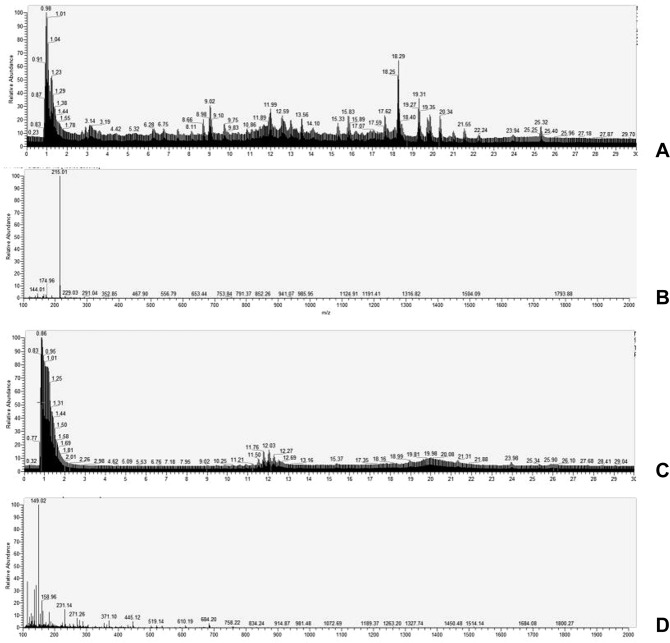
UPLC-MS results of SQFZ injection ((**A**,**B**) is the results of negative ionization mode; (**C**,**D**) is the results of positive ionization mode).

The UPLC-MS results were shown in Fig. 1. After compared the results with database and other researches^17–21^, a total of 34 ingredients were achieved and used in network analysis.

The ingredients found in UPLC-MS results are Lobetyolin, Astragaloside I, Astragaloside II, Astragaloside III, Astragaloside IV, Isoastragaloside I, Isoastragaloside II, AcetytastragalosideI, conifers glycosides violet, Syrigin, Glucosyringic acid, Fructose, Guanosine, Adenosine, vanillic acid, hexyl-beta-Locust glycoside, hexyl-beta-gentiingentiomarin, Calycosin, calycosin7-O-beta-D-glucopyranoside, Formononetin, Formononetin-7-O-beta-D-glucopyranoside, (6a,11aR)-9, 10-Dimethoxysandalane-3-O-beta-D-glucopyranoside, 5-hydroxymethylfurfural, (3 R)-(−)-2′-hydroxy-3′,4′-dimethoxyisoflavane7-O-beta-D-glucopyranoside, (+)-Syringaresinol-4-O-beta-D-glucopyranoside, Pratensein7-O-beta-D-glucopyranoside, glycyrrhizinD, Syringaresinol glycoside, coniferin, (3 R)-7,2′dihydroxy-3′,4′-dimethoxyisoflavane, brachyoside B, Creoside IV, (6aR,11aR)-vesticarpan and (Z)-cinnamicacid 8-O-β-D-glucopyranoside.

### cC-cT network

**Figure 2:**
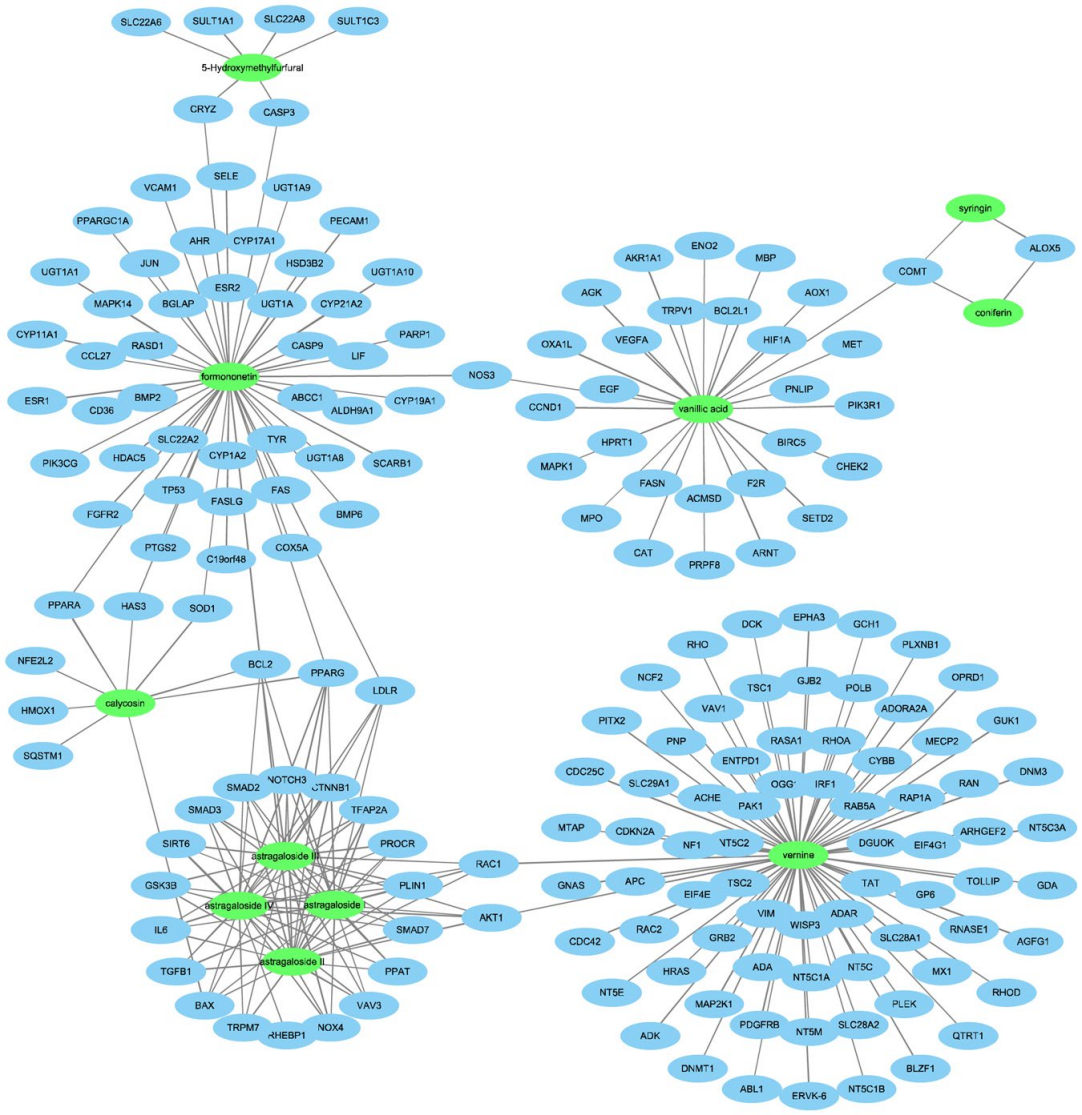
Network of the 11 compounds in SQFZ injection predicted to have 182 candidate targets.

The target prediction of SQFZ injection was based on ingredient determination in 3.1. After searched in Genecards database, 11 of 34 ingredients were found having known targets. The network of SQFZ injection (Fig. 2) comprised 193 nodes (11 compounds and 182 targets). The nodes of compounds of SQFZ injection and their candidate targets were shown in green color and blue color, respectively.

The edge interactions indicate that most of the candidate targets are hit by only one candidate compounds, but some can be modulated by multiple compounds.

**Figure 3:**
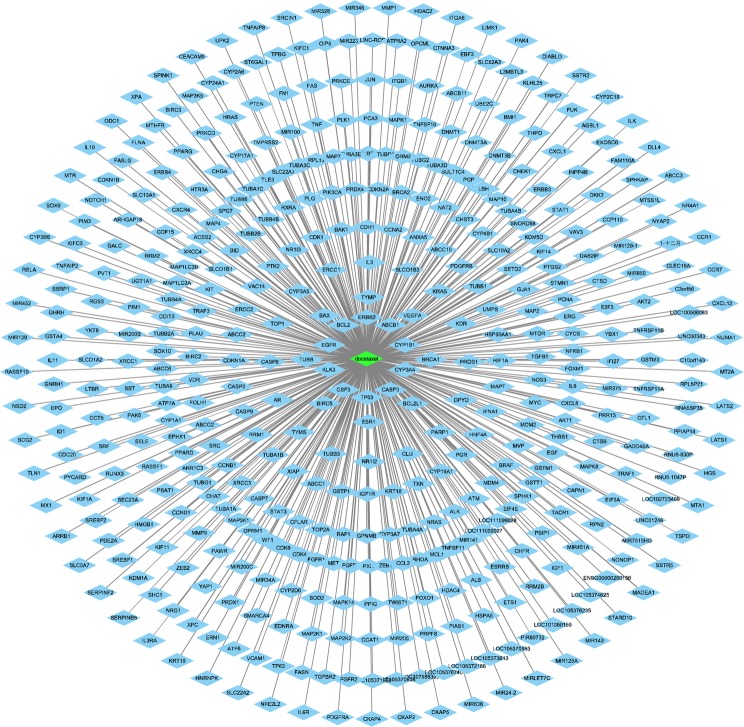
Network of docetaxel predicted to have 452 candidate targets.

The network of docetaxel (Fig. 3) comprised 452 nodes. The node in green color is docetaxel, and those in blue colour are its candidate targets. The length of edges between targets and docetaxel are related to their relevance score. The targets in the outer circle show less interactions with docetaxel than those in inner area.

### C-pT-D network

**Figure 4:**
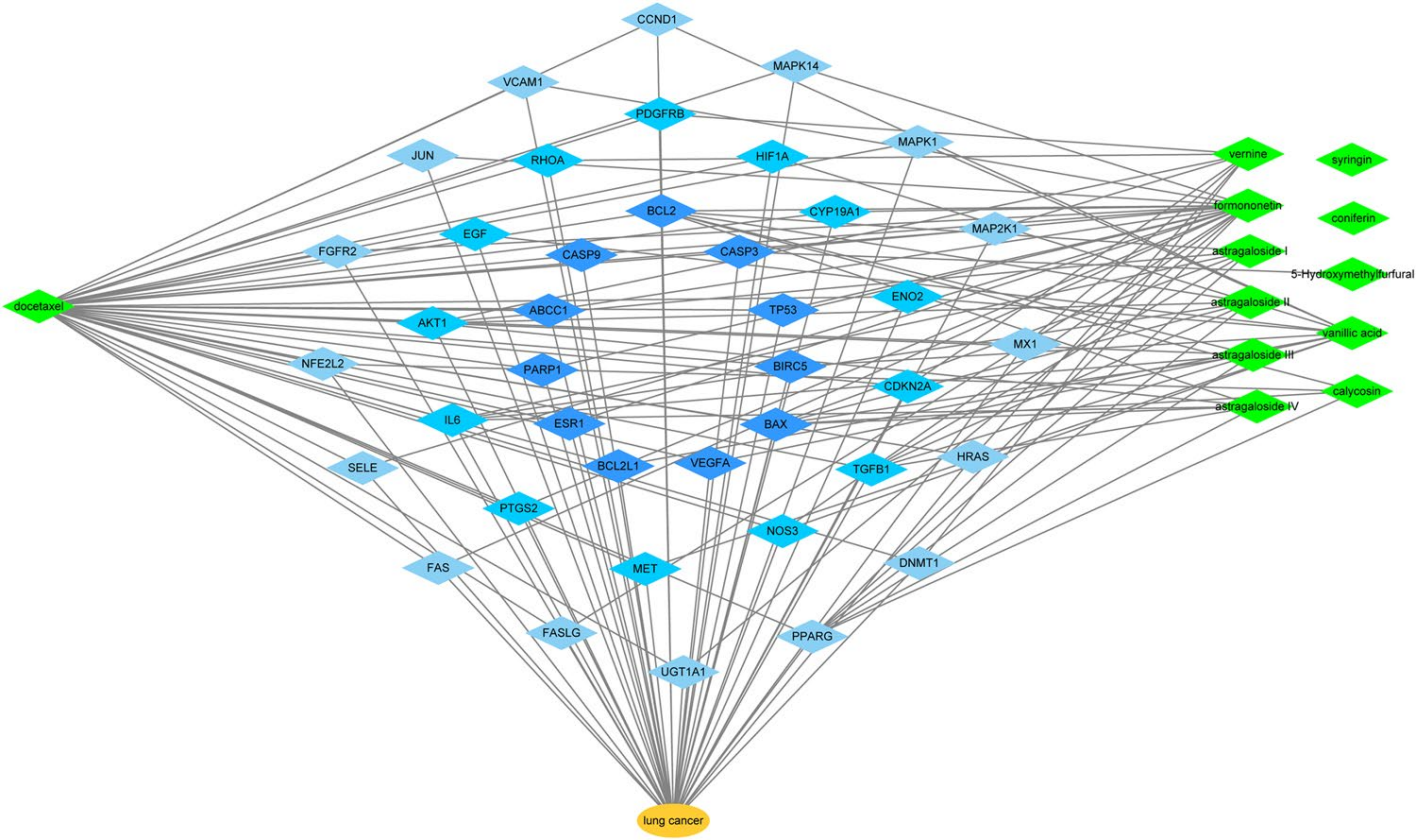
Network of docetaxel, SQFZ injection and lung cancer predicted to have 40 common potential targets.

The relationships between docetaxel, SQFZ injection and lung cancer were investigated by connecting targets related to docetaxel, SQFZ injection and lung cancer at the same time (Fig. 4). The remaining 40 targets were used to build a compound-potential targets-disease (C-pT-D) network. The network represents a global view of docetaxel (green nodes), SQFZ injection (green nodes), lung cancer (dark yellow nodes)and their potential targets (blue nodes). We further investigated the relevance score of each node and sequenced them with color depth. The targets in light blue color show fewer interactions with lung cancer than those in dark blue color. For the node in same colour, the one in right above shows the highest relevance score with lung cancer and the scores become lower in clockwise. Therefore, we chose the targets with highest scores, BCL2 and CASP3, to conduct the *in vivo* and *in vitro* experiment.

### Pharmacological verification on network analysis

#### *In Vivo* Experiment

**Figure 5:**
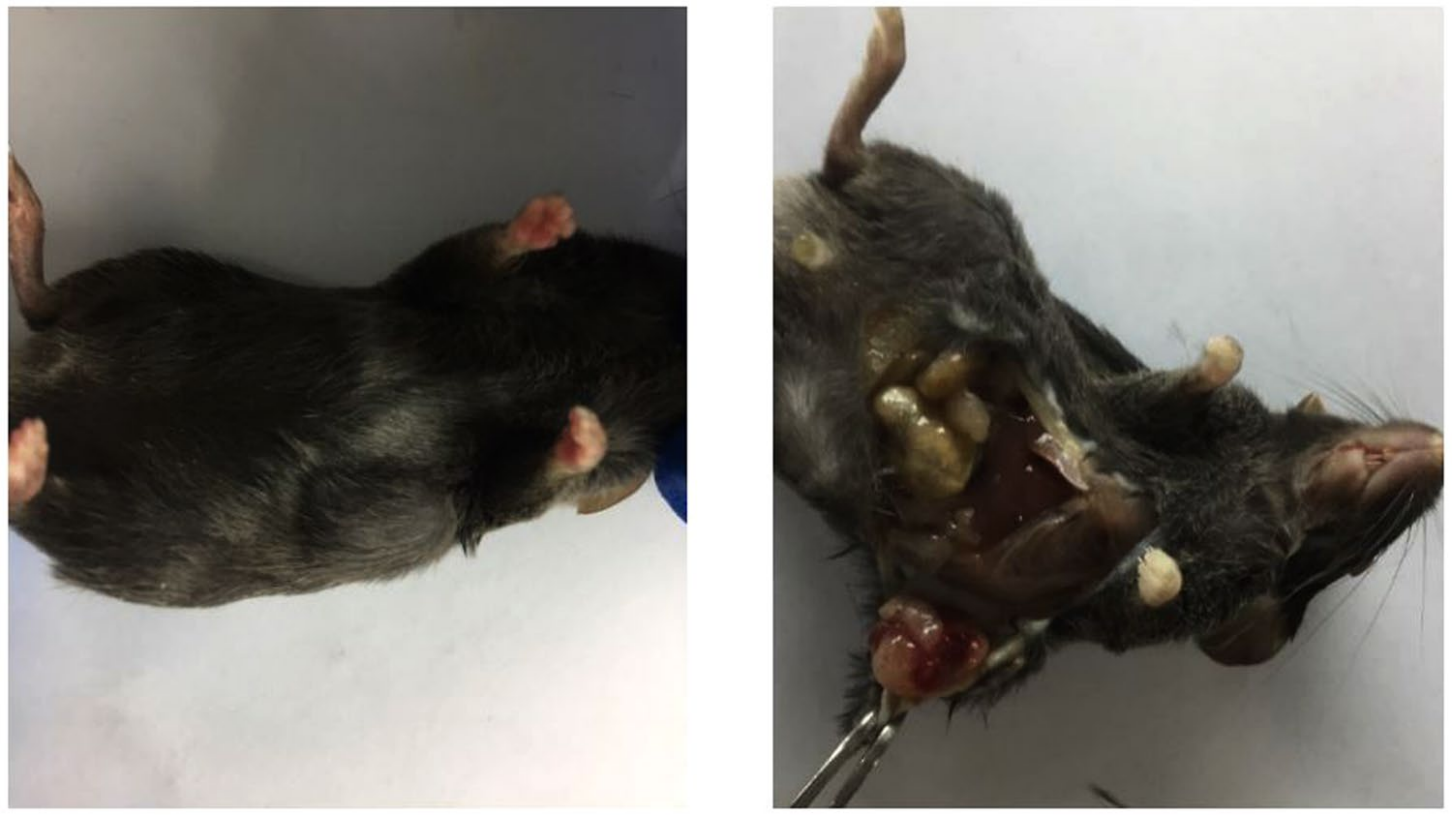
Mice subcutaneous lung cancer model.

Establishment of subcutaneous lung cancer model: The regular condition of mice was observed after cancer cell injection. The coat colour and mental state had changed two weeks after injection. Tumour can be seen under left axillary region (Fig. 5).

Thymus index and spleen index: Thymus index and Spleen index represent the ratio of thymus/body and Spleen/body, respectively.

**Figure 6:**
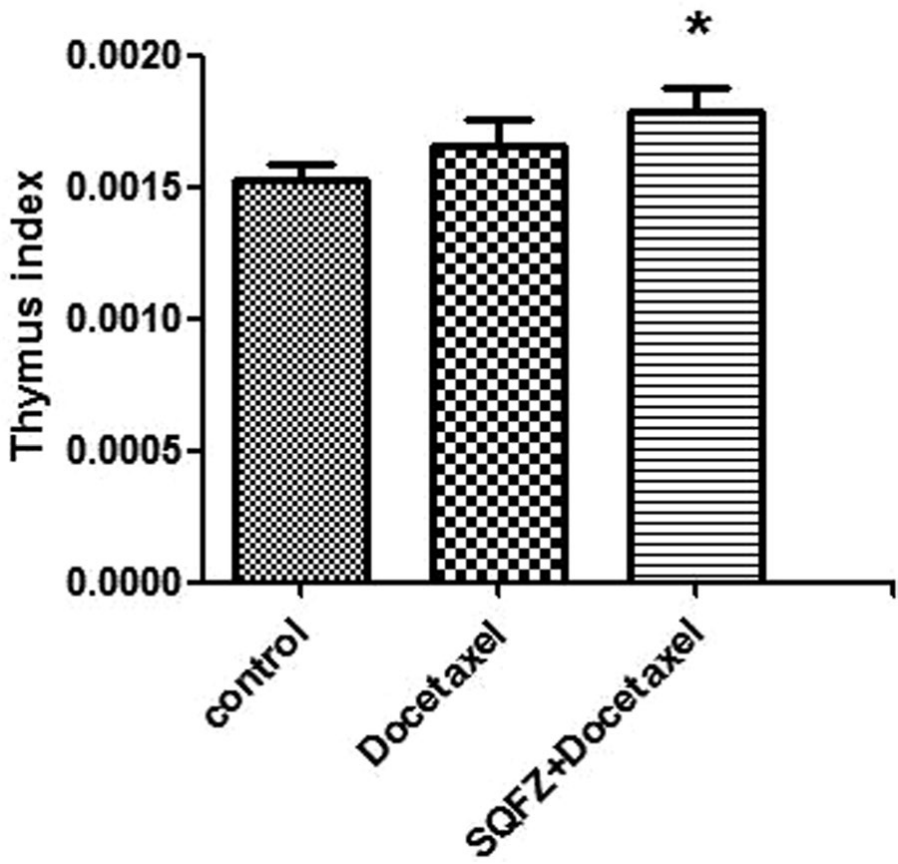
Result of thymus index. *p < 0.05 compared to control group.

The main function of thymic is producing T lymphocytes and thymosin secretion. SQFZ injection + docetaxel group shows the highest Thymus index in Fig. 6. However, there is no statistical significance between each group, which indicates that SQFZ injection in combination with docetaxel may perform a stronger cellular immunity.

**Figure 7:**
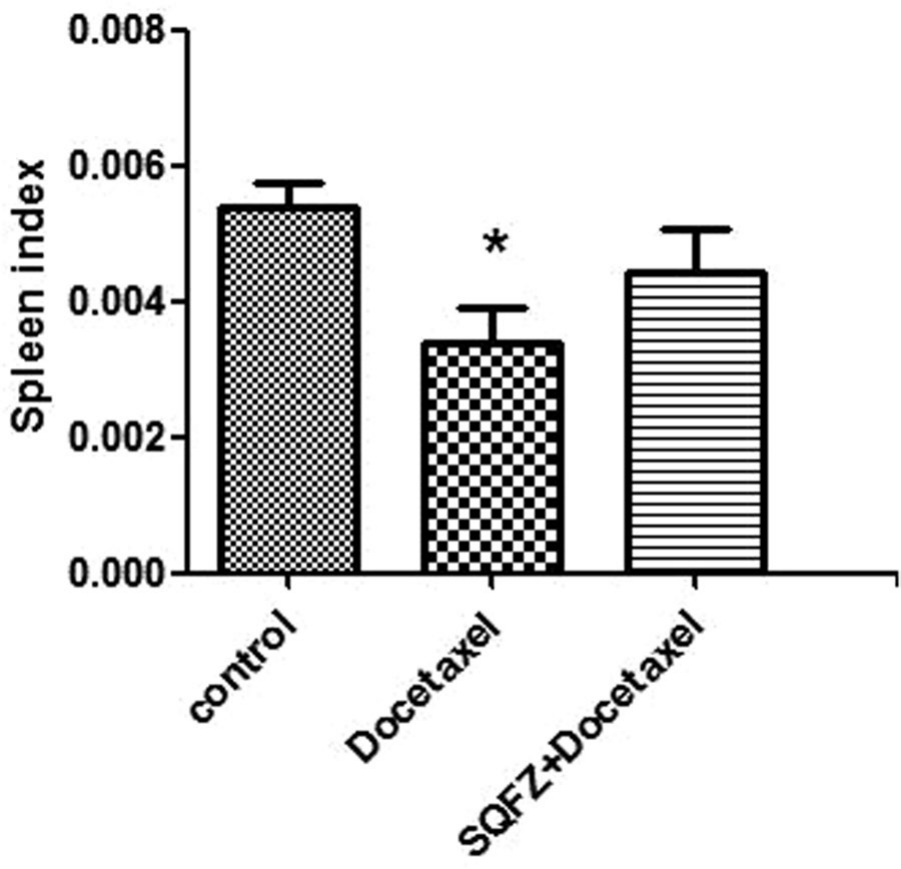
Result of spleen index. *p < 0.05 compared to control group.

The spleen contains abundant lymphocytes and macrophages,most of them are B lymphocytes. The wight and function of spleen associate with number of B lymphocytes.The spleen index of each group shows in Fig. 7.Docetaxel group has a lower spleen index compare with control group. Meanwhile, combining with SQFZ injection could promote the index which indicates that SQFZ injection in combination with docetaxel may perform a stronger humoral immunity.

**Figure 8:**
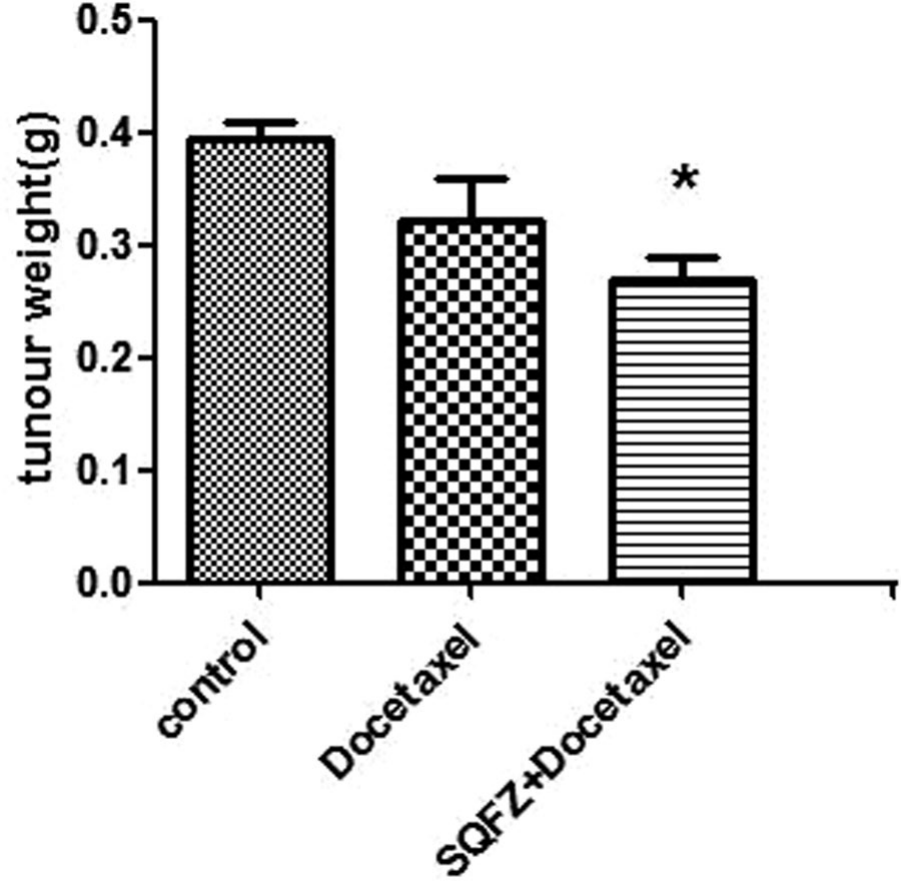
Result of tumour weight. *p < 0.05 compared to control group.

Inhibition rate of tumor weight: The Inhibition rate of tumor weight shows in Fig. 8. Comparing with control group and docetaxel group, SQFZ + docetaxel group shows a significant Inhibition of tumour. Which indicates that SQFZ injection might could improve the efficacy of docetaxel.

**Figure 9:**
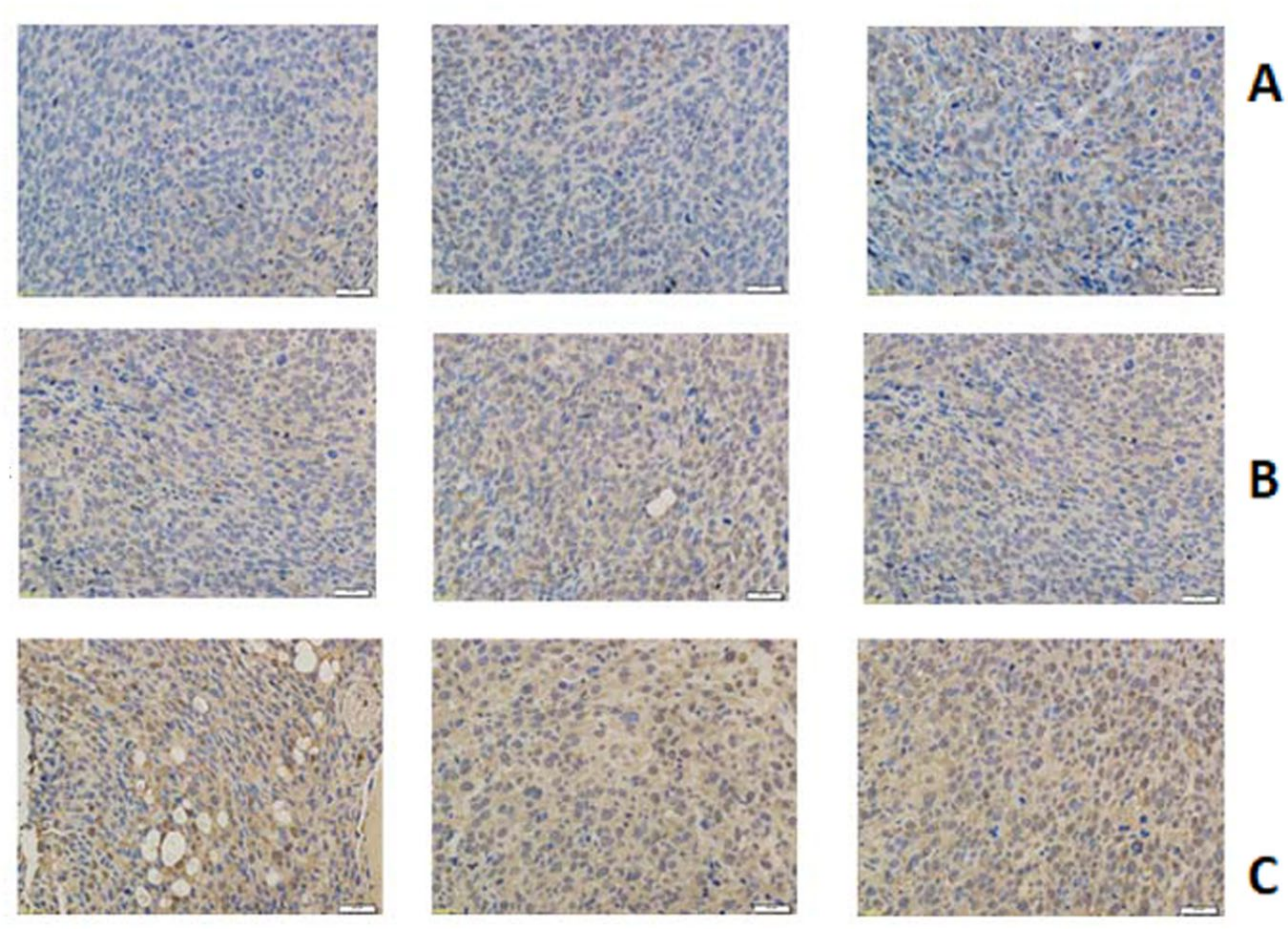
Immunohistochemical staining results of each group (A: control group; B: docetaxel group; C: SQFZ + docetaxel group).

**Figure 10:**
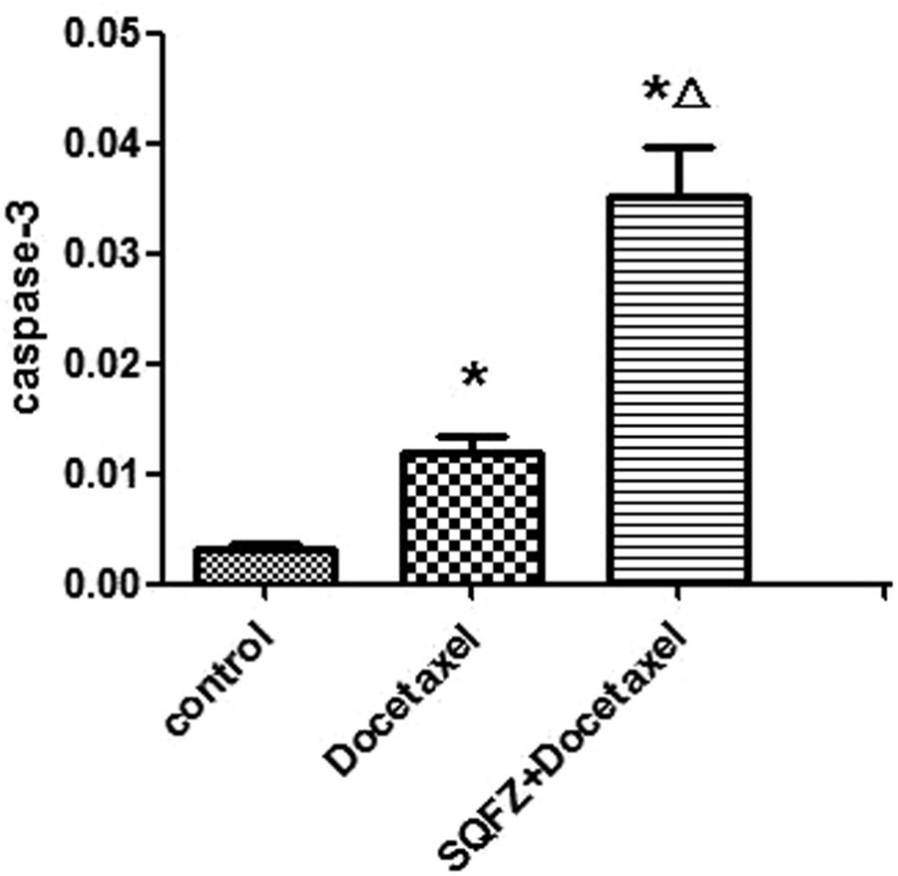
Result of caspase-3 activity. *p < 0.05 compared to control group. ^△^p < 0.05 compared to docetaxel group.

Immunohistochemical Staining: The result of immunohistochemical staining shown in Fig. 9, yellow colour stand for caspase-3 expression, which also indicates the anti-tumour effect. Compared with control group, docetaxel group exhibited a darker yellow colour. Meanwhile, the color of SQFZ + docetaxel group changed most among these groups. After analyzed average optical density, SQFZ + docetaxelgroup showed a significant caspase-3 activity comparing with control group and docetaxel group (Fig. 10).

**Figure 11:**
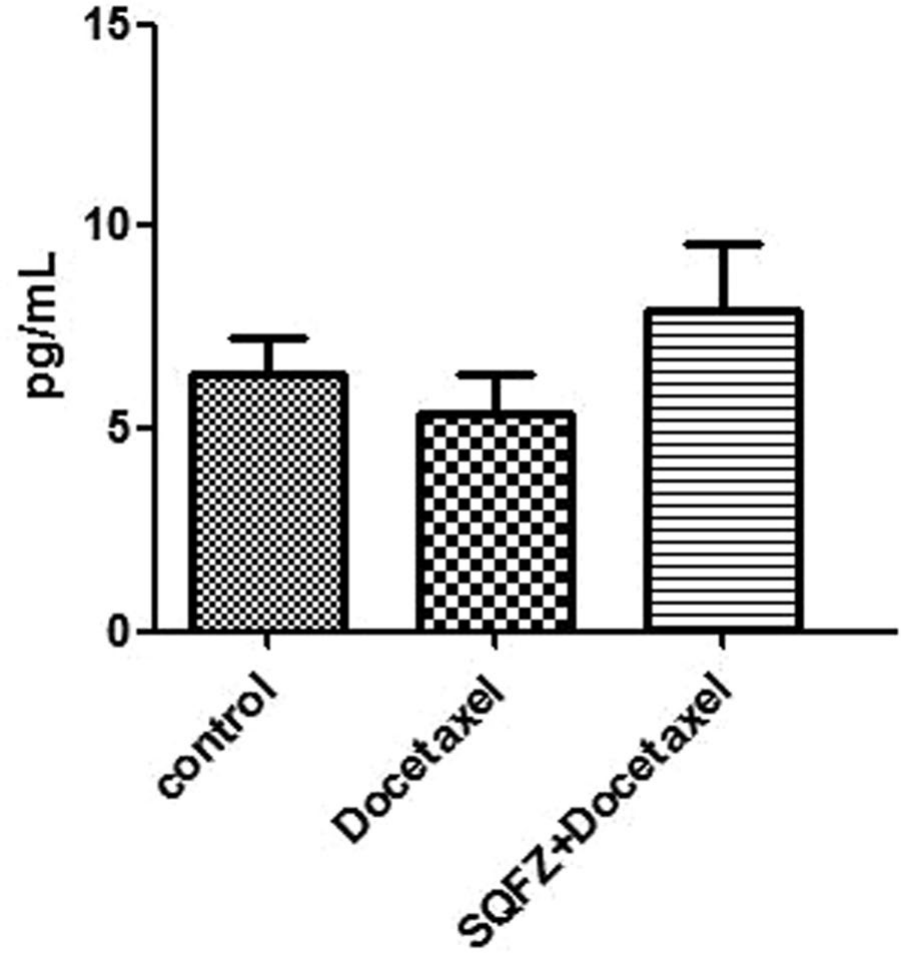
Result of IL-2 activity.

Enzyme-Linked Immunosorbent Assays (ELISA) of IL-2: IL-2 is an immune factor. The result of ELISA shows in Fig. 11. IL-2 activity of docetaxel group has decreased compared with control group, which means docetaxel might affect the immune function of mice. On the other hand, comparined with the other 2 groups, SQFZ + docetaxel group showed the highest IL-2 activity. This result corresponds with the findings in other researches that SQFZ injection could increase immunopotency of the mice with cancer.

### *In Vitro* Experiment

#### Western Blot Analysis

**Figure 12:**
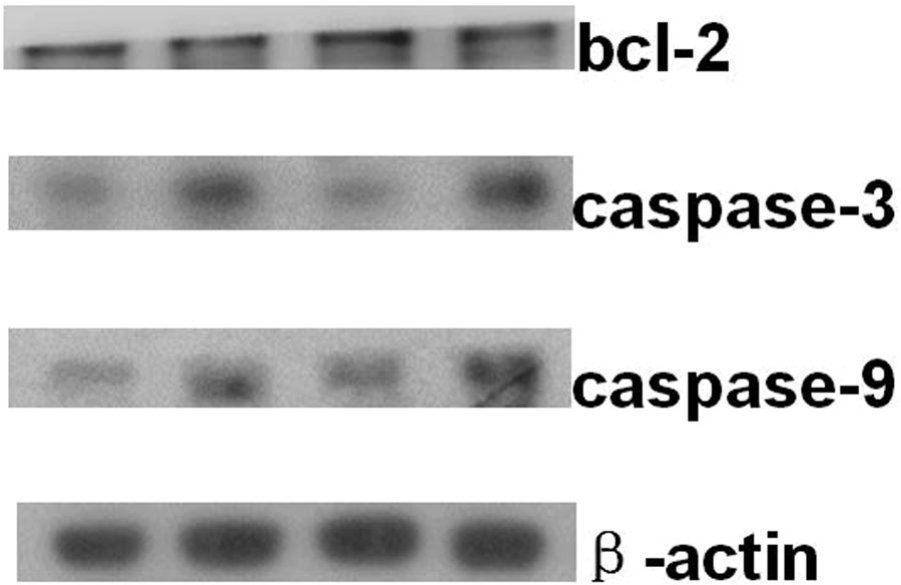
Results of western-blot analysis.

**Figure 13:**
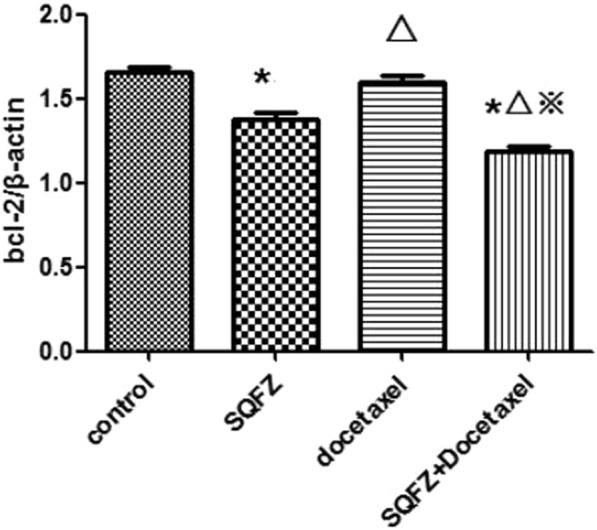
Result of BCL-2 activity *p < 0.05 compared to control group; ^Δ^p < 0.05 compared to SQFZ group; ^※^p < 0.05 compared to docetaxel group.

**Figure 14:**
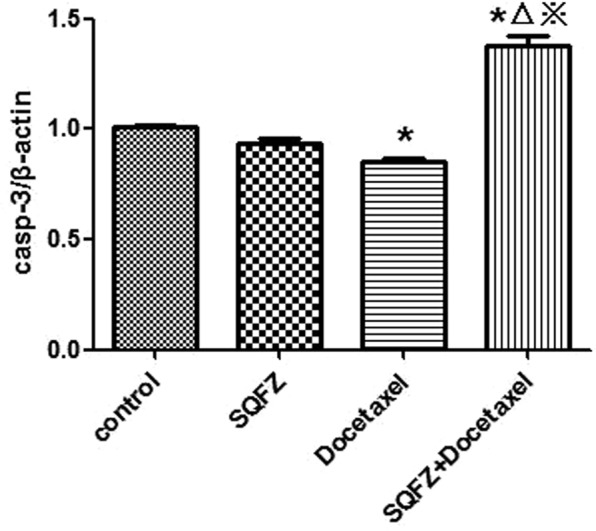
Result of caspase-3 activity *p < 0.05 compared to control group; ^△^p < 0.05 compared to SQFZ group; ^※^p < 0.05 compared to docetaxel group.

**Figure 15:**
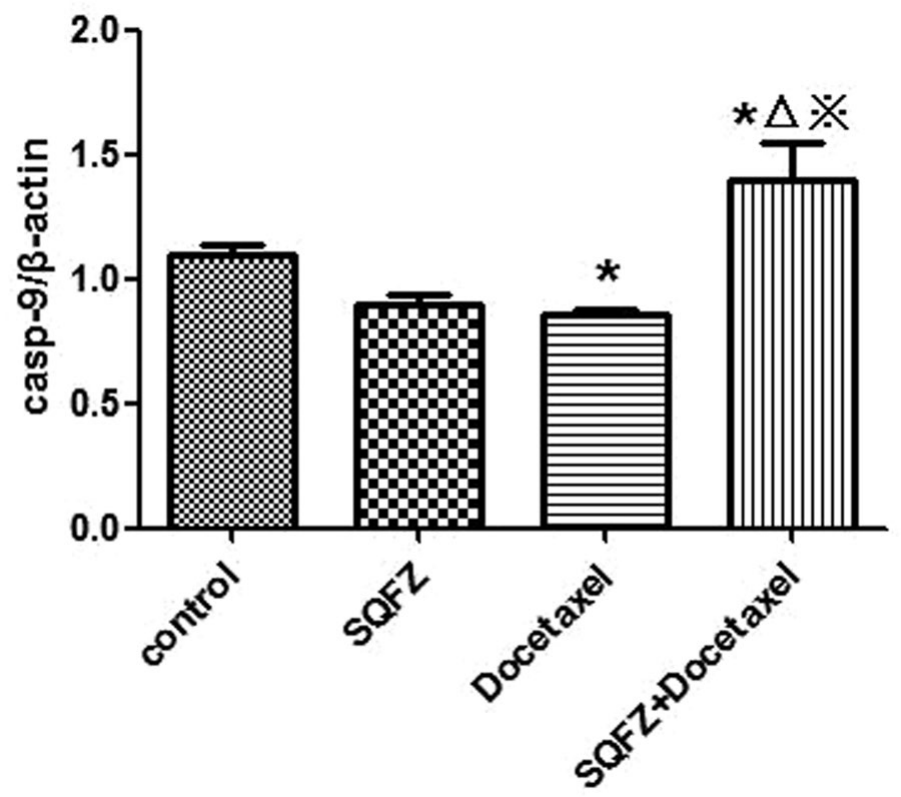
Result of caspase-9 activity. *p < 0.05 compared to control group; ^△^p < 0.05 compared to SQFZ group; ^※^p < 0.05 compared to docetaxel group.

In order to clarify the mechanism of SQFZ injection in combination with docetaxel on lung cancer, caspase-9 was also measured except for bcl-2 and caspase-3, because it is also a key target in network analysis and has a close connection with caspase-3 in apoptosis signaling pathway, except for bcl-2 and caspase-3. The results of western blot analysis were shown in Figs 12–15. Compared with control group, the caspase-3 and caspase-9 activity of docetaxel group and SQFZ group have decreased, however, the activity of SQFZ + docetaxel group has increased significantly. And the bcl-2 activity of SQFZ group and SQFZ + docetaxel group has decreased significantly compared with control group and docetaxel group. Which indicates that SQFZ injection combined with docetaxel could induce apoptosis in A549 cell.

## Discussion

Lung cancer is one of the most common and lethal malignancies around the world. Chemotherapy is necessary method during the whole curative process. However docetaxel, regarded as the main chemotherapy for lung cancer, has some limits including high recurrence rate and side effects such as immune injury, gastrointestinal injury and so on. More and more clinicians aim to reduce the side effect and recurrence rate through medicine combination. Herb medicine has been proven to have such clinical effects. SQFZ injection, a purified extraction of herb medicine, has been applied as chemotherapeutic adjuvant and turned out to have obvious effect. The major ingredients of SQFZ are extracted from odonopsis pilosula and astragalus membranaceus, which could improve immune function^22–25^. In this study, we combined SQFZ injection with the first-line chemotherapy drug docetaxel to find out the anti-tumour mechanism of drug-drug combination. Meanwhile, we conducted experiments to testify SQFZ injection’s potential pharmacology action to ameliorate the immune damage caused by docetaxel.

In order to find the main ingredients of SQFZ injection, we first conducted UPLC-MS analysis to acquire its mass spectrogram. Then we intersected exact molecular weights from mass spectrogram and the ones from database and literature to verify the possible ingredients of SQFZ injection. Based on the verified ingredients, we applied a network pharmacology analysis to predict the potential effect-enhancing targets of SQFZ injection and docetaxel on lung cancer. The result indicates that the main compounds in SQFZ injection, such as 5-Hydroxymethylfurfural, astragaloside, formononetin and calycosin, may have pharmacological action for lung cancer via inducing apoptosis of tumour cells. Then we chose several key targets of apoptosis for *in vivo* and *in vitro* experiments. Moreover, as reported in several clinical studies, immunity improvement might be a major effect of SQFZ injection, so thymus index, spleen index and IL-2 were further investigated to evaluate immunity changes^26^.

The results of *in vivo* and *in vitro* experiments implied that SQFZ injection combining with docetaxel could reduce the tumour weight, raise the thymus index and spleen index in mice, which means combining with SQFZ injection, the anti-tumour effect has been enhanced and the decrease of immune function caused by docetaxel has been alleviated. Moreover, the combination of two drugs could induce the activity of caspase-3 in both A549 cell and mice. Also, it could induce the activity of caspase-9 and inhibit the activity of bcl-2 in A549 cell. Several researches shows that overexpression of caspase-3 could induce apoptosis^27^. Bcl-2 and caspase-9 are the upstream protein of caspase-3. There is a positive correlation between bcl-2 and caspase-9 and a negative correlation between caspase-9 and caspase-3^27^. Therefore, the anti-tumour mechanism of drug-drug combination is to induce apoptosis through up-regulating the activity of caspase-3 and caspase-9 by inhibiting the activity of bcl-2.

Besides, based on the results of *in vivo* and *in vitro* experiments, we can also use C-pT-D network (Fig. 4) to find out the ingredients in SQFZ injection linking to BCL-2, CASP3 and CASP9. These ingredients are astragaloside I, astragaloside II, astragaloside III, astragaloside IV, formononetin and calycosin, which might be the pharmacology active ingredients of SQFZ injection that induce apoptosis in tumour.

Moreover, the *in vivo* experiment showed that spleen index and IL-2 were reduced by only docetaxel administration while enhanced when in combination with SQFZ injection. This result indicates that SQFZ injection could ameliorate the immune damage caused by docetaxel.

## Conclusion

The anti-tumour mechanism of drug-drug combination is to induce apoptosis through up-regulating the activity of caspase-3 and caspase-9 by inhibiting the activity of bcl-2. This regulation process is more significant than docetaxel alone. Also, SQFZ injection could alleviate the reduce of spleen index and IL-2 caused by docetaxel in mouse. Therefore, the combination of SQFZ injection and docetaxel may be served as rational drug to play a synergistic effect. The combination might be a wise treatment strategy for lung cancer rather than docetaxel alone.
